# Chromosome Pairing: A Hidden Treasure No More

**DOI:** 10.1371/journal.pgen.1002737

**Published:** 2012-05-24

**Authors:** Giovanni Bosco

**Affiliations:** Department of Molecular and Cell Biology, University of Arizona, Tucson, Arizona, United States of America; The University of North Carolina at Chapel Hill, United States of America

Many generations of biologists have been intrigued by the myriad structures that eukaryotic chromosomes can adopt and have questioned how their form relates to function [1], [2]. One organizational state that chromosomes adopt is pairing in a homology-dependent manner [3]–[8]. Although homolog pairing in meiosis has been extensively studied and is important for chromosome segregation, pairing of homologs in somatic cells is less well understood. In this issue of *PLoS Genetics*, Joyce et al. report the first comprehensive RNAi screen of genes regulating somatic chromosome pairing in Drosophila [9]. This study finally unlocks the treasure trove of Drosophila somatic chromosome pairing, sets the stage for much deeper mechanistic investigations, and most importantly, points to new avenues for understanding the functional significance of homologous chromosome pairing.

Drosophila presents a unique opportunity for identifying molecular regulators that establish, maintain, and antagonize homolog pairing because its homologous chromosomes are almost always paired in somatic cells. Metz described somatic cell homolog pairing in 1916 [10], while Painter first described polytene chromosomes in 1933 [11]—polytene chromosomes are found in some polyploid cells where many copies of homologous chromosomes and chromatids are paired along their lengths. Despite these early descriptions of chromosome pairing, many fundamental questions regarding homolog pairing still remain unanswered: Is meiotic homolog pairing mechanistically similar to pairing in somatic cells? Is pairing of homologous sequences in the context of polytene chromosomes similar to somatic or meiotic homolog pairing? In the absence of recombination- and meiosis-specific synaptonemal complex proteins, how do homologous sequences find each other in somatic cells? Are there negative regulators of pairing? Are there genomic regions or chromatin states that pair more efficiently than others? Most importantly, what is the biological relevance of homolog pairing in somatic cells? The answers to these questions have eluded us for almost a century because of limitations in cytological tools for measuring pairing and genetic tools for perturbing pairing dynamics.

**Figure 1:**
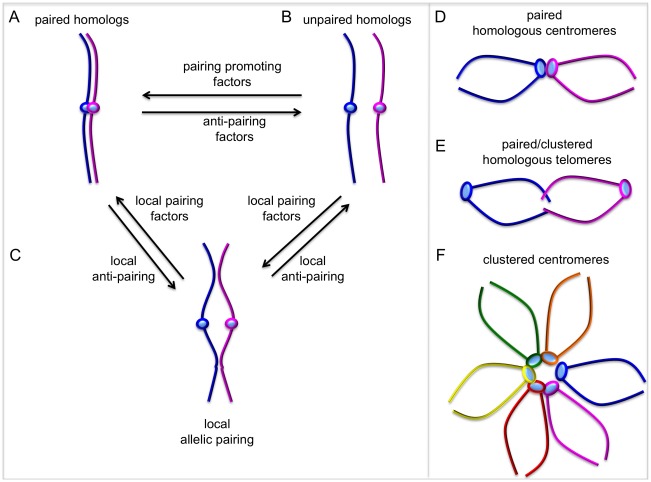
Dynamic chromosome pairing. The term “pairing” describes the spatial juxtaposition of entire homologous chromosomes, allelic sequences, and/or homologous sequences at non-allelic locations. (A, B) Homologous chromosomes can exist as paired throughout their entire length and the relative activities of pairing and anti-pairing factors determine the degree of global chromosome pairing. (C) Local pairing and anti-pairing factors can affect pairing status of specific genes or chromosomal regions. Local “pairing centers” can nucleate global pairing, but it is unclear whether factors regulating local pairing are different from global pairing factors. (D–F) In some cases, centromeres or telomeres from homologs can pair or cluster with non-homologous chromosomes, while there is no pairing along chromosome arms.

Recent evidence has raised the exciting possibility that both pairing and anti-pairing forces may act on chromosomes to regulate the spatial juxtaposition of homologous sequences (Figure 1). Two previous studies in Drosophila identified *Suppressor of Hairy Wing* (*Su(Hw)*) and *Topoisomerase II* as pairing promoting factors [12], [13]. In a third study, the Kleisin subunit of condensin II, *Cap-H2*, was shown to be necessary and sufficient to antagonize pairing of homologs in the context of polytene chromosomes [14]. *Cap-H2* mutant Drosophila males have chromosome unpairing defects in meiosis I, also providing evidence for a Cap-H2 anti-pairing activity [15]. Until now, the dearth of molecular models for somatic pairing has been mainly due to this paucity of “pairing” and “unpairing” factors.

The ability to perturb homolog pairing by RNAi depletion [13], combined with FISH and high-throughput technology, has now made it possible to interrogate entire genomes and ask, nearly 100 years after Metz's initial description, “what genes regulate homologous chromosome pairing in somatic cells?” Joyce et al. [9] use a novel combination of whole genome RNAi, high-throughput imaging, and DNA FISH that represents a tremendous effort. This work is a significant advance because it provides an extensive “parts list” of mostly novel factors affecting pairing. In their elegant RNAi screen, Joyce et al. report 40 new pairing promoting genes (where previously we knew of two) and 65 new anti-pairing genes (where previously we knew of only one). Interestingly, identification of genes affecting pairing of heterochromatic or euchromatic regions, but not both, supports the idea that pairing of different chromatin domains may be regulated in different ways.

The pairing and anti-pairing genes code for cell cycle, protein turn-over machinery, and chromatin proteins, among others. Previous studies suggested that cell cycle regulation and chromosome pairing are related by showing that entry into S-phase and G2/M disrupt pairing [13], [16], [17]. It is also likely that some cell cycle genes directly regulate pairing or may even monitor pairing status. For example, if allelic or homolog pairing in G1 is important for specific gene expression states, then one might imagine that in cycling cells pairing may be preserved through multiple mitotic chromosome condensation/decondensation cycles. Alternatively, if DNA replication and chromosome compaction forces disrupt pairing, then G1-specific regulators may be required to re-establish pairing. Now that we know which cell cycle genes affect pairing, the next challenge is to understand how they function in pairing dynamics. Of the protein turn-over genes that promote pairing, the Slimb ubiquitin ligase is of particular interest. This is because the authors show that Slimb-RNAi disruption of pairing is rescued by RNAi depletion of condensin II genes. This again points to a condensin II anti-pairing activity. However, a direct link between condensation and pairing is yet to be determined. That Slimb may target one or more anti-pairing factors while components of the Anaphase-Promoting Complex (APC) promote pairing suggests a still more complex layer of pairing regulation that ties protein turn-over machinery back to cell cycle regulation. It will be of great interest to determine the direct targets of Slimb- and APC-mediated protein turn-over and how these targets function in pairing.

Perhaps the most exciting broad conclusions from this study are that chromosome pairing is much more complicated and dynamic than anyone had anticipated, and that an abundance of “pairing promoting” and “anti-pairing” factors provide opposing forces. The authors suggest that the degree of homolog pairing in somatic cells, at the gene level and at the whole chromosome level, is likely determined by the relative activities of pairing and anti-pairing factors (Figure 1). It is noteworthy that many of the genes revealed in this study also have orthologs in other species, including humans. With this new pairing parts list it will be possible now to ask how homolog pairing in different species is regulated, and more importantly, it will lead to new studies seeking to understand the biological relevance of somatic pairing in different species.
